# Comparative efficacy and acceptability of traditional Chinese medicine for adult major depression: A protocol for network meta-analysis

**DOI:** 10.1097/MD.0000000000023199

**Published:** 2020-11-20

**Authors:** Yuze Shao, Kai Wang, Zhenyuan Jiang, Xiaowen Yu, Wenrong An, Yue Han, Zhonglin Wang

**Affiliations:** a Shandong University of Traditional Chinese Medicine; b Shandong Provincial Hospital Affiliated to Shandong First Medical University; c Affiliated Hospital of Shandong University of Traditional Chinese Medicine, Jinan, Shandong, China.

**Keywords:** traditional Chinese medicine, major depression, protocol, systematic review

## Abstract

**Background:**

Major depression disorder (MDD) is a severe health threat characterized by persistent depression, loss of interests, lack of initiative, and even suicidal tendencies. Traditional Chinese medicine (TCM) is well tolerated and effective in treating adult MDD. However, research on the evaluation of efficacy and acceptability of different TCM strategies for adult MDD is insufficient. Consequently, it is high time to evaluate the efficacy of TCM strategies for adult MDD. Meanwhile, the acceptability of different TCM strategies is worth exploring.

**Methods:**

Comprehensively and systematically retrieve the literature in PubMed, Cochrane Library, Web of Science, Embase, China National Knowledge Infrastructure Database (CNKI), Wanfang Database, China Science and Technology Journal Database (VIP), and Chinese BioMedical Literature Database (CBM). The literature search will focus on randomized controlled trials (RCTs) with TCM in adult MDD. Two reviewers will search the literature and extract relevant data back-to-back. Once mismatched outcomes appear, arbitration will be conducted by a third reviewer. Based on the Bayesian framework, data analysis is carried out with STATA and WinBUGS software. Heterogeneity, transitivity, consistency test, bias risk assessment, subgroup and sensitivity analysis, evidence quality evaluation will be performed accordingly.

**Results:**

The efficacy and acceptability of different TCM strategies for adults with MDD will be compared and sorted.

**Conclusion:**

The study will facilitate the treatment options of adults MDD according to the supporting evidence.

**INPLASY registration number:**

INPLASY2020100028.

## 1 Introduction

As a kind of severe emotional disorder, major depressive disorder (MDD) is characterized by low mood, lack of pleasure, and loss of interest, often accompanied by anxiety, cognitive impairment, psychomotor retardation, and even suicidal tendencies.^[1,2]^ Social and cultural factors have always had a significant role in the pathogenesis of MDD. However, the 12-month prevalence of MDD is almost the same in developed and non-developed countries, with the former being 5.5% and the latter being 5.9%.^[3]^ Therefore, MDD is not a simple consequence of an excessively fast pace of life or poor living conditions. Depression has sex-specific genetic effects. The incidence of depression is 13% in males, and 21% in females, which is nearly double that of men.^[4,5]^ Besides, the pathogeneses of MDD is also associated with neuroendocrine, immunity, gut microbiota, etc.^[6–8]^ Depression is the most common mental disorder; about 350 million people worldwide suffer from MDD.^[9]^ The 12-month prevalence and lifelong morbidity are 6.6% and 16.2%, respectively, indicating that almost 1 in 5 people will suffer from MDD attacks at some point in their life.^[3,10]^ As of 2013, mental health conditions is added to the leading causes of disability list due to its destructive impacts, with a prediction that MDD will become the first cause ranked for disability by 2025.^[11]^ The tremendous healthcare costs of depression globally present a concern. It is estimated that the global costs of depression and anxiety reach $1.15 trillion per year.^[12]^

Currently, antidepressants such as trazodone and monoamine oxidase inhibitors (MAOIs), selective serotonin reuptake inhibitors (SSRIs) and serotonin-norepinephrine reuptake inhibitors (SNRIs) are widely used to treat MDD.^[13]^ However, there are still many debates about the therapeutic benefits for short term administration and potential risks for long term administration of these drugs. It is reported that approximately 30% to 50% of patients with MDD lack response to SSRIs.^[14]^ Chronic TCAs administration in patients with ischemic heart disease (IDH) and depression can lead to multiple cardiovascular adverse events, including heart rate acceleration, postural hypotension, atrioventricular block, and even increase the risk of cardiac morbidity.^[15]^ Therefore, in clinical practice, clinicians need to make the most suitable choice for each patient in a wide range of antidepressants based on their rich clinical experience.

The network meta-analyses provide the possibility for the comparison of multiple treatment strategies, and researchers have used this method to conduct some studies on drug and non-drug treatment of MDD. The efficacy and acceptability of 21 antidepressants for adult MDD were summarized in a network meta-analysis. Research indicated that 21 antidepressants were better than placebo in efficacy, and Amitriptyline, Mirtazapine, and Duloxetine were the top 3 in response rate; in terms of dropout rate, only Agomelatine and Fluoxetine were lower than placebo.^[16]^ Another network meta-analysis which compared efficacy and acceptability of non-surgical brain stimulation for MDD showed better effects of bitemporal electroconvulsive therapy and priming transcranial magnetic stimulation; in terms of acceptance, the included non-surgical brain stimulation methods were the same as that of the sham therapy.^[17]^ There is also a network meta-analysis on the use of exercise therapy to treat depression in the elderly. The study confirmed the antidepressant effect of exercise, and mind-body exercise achieved an optimal degree of improvement on depressive symptoms.^[18]^ Different network meta-analysis can help doctors make clinical decisions to a certain extent. However, there is currently no evaluation of the effectiveness and acceptability of traditional Chinese medicine (TCM)for adult MDD. TCM has done a lot of basic research on the treatment of depression. Shexiang Baoxin pill has been verified for anti-depression through inhibiting hypothalamic-pituitary-adrenal (HPA) axis hyperfunction, regulating the release of monoamine neurotransmitters and promoting the secretion of neurotrophins.^[19]^ Noteworthy, as a medicine for treating heart disease, Shexiang Baoxin Pill may be especially appropriate for patients comorbid with cardiac conditions and depressive disorder. Xiaoyao Power can improve depressive-like behaviour through modulating gut microbiota and intervening in brain metabolism.^[20,21]^ Kaixin Power can relieve depression symptoms. The mechanism has been shown to be associated with inducing the expression of neurotrophic factors and promoting neurogenesis.^[22]^ A wealth of clinical evidence demonstrates the efficacy of TCM strategies for MDD. However, the differences in efficacy and acceptability of different TCM strategies on adults MDD is worthy of further study.

Therefore, the comparison of the efficacy and acceptability of western medicine alone vs combined TCM and western medicine for adult MDD is in great request. In the current study, we will present an analysis of the efficacy and acceptability by using a Bayesian approach to provide practical clinical reference value.

## 2 Methods

Complete the protocol registration from the International Platform of Registered Systematic Review and Meta-analysis Protocols (registration number: INPLASY2020100028; URL = https://inplasy.com/inplasy-2020-10-0028/). In addition, we will perform the NMA protocol based on the Preferred Reporting Items for Systematic Review and Meta-Analysis Protocols (PRISMA-P) guidelines.^[23]^

### 2.1 Search strategy

Cochrane Library, PubMed, Web of Science, Embase, CNKI Database, Wanfang Database, VIP Database, and CBM Database are the screened target database. The search time is limited from the establishment of each database to September 2020. No restriction will be applied for language and publication. The search strategy is implemented by a combination of thesaurus terms (Medical Subject Headings in Pubmed) and free-text terms. Table 1 shows the detailed search strategy of the PubMed database. Moreover, the reference of selected articles will also be evaluated. After the retrieval, all the literature will be imported into EndnoteX9 for literature management and analysis.

**Table 1:** Search strategy for Pubmed.

No.	Search item
#1	Depressive Disoder, Major[MeSH]
#2	Depressive Disorders, Major [Title/Abstract] OR Major Depressive Disorders [Title/Abstract] OR Major Depressive Disorder [Title/Abstract] OR Paraphrenia, Involutional [Title/Abstract] OR Involutional Paraphrenia [Title/Abstract] OR Involutional Paraphrenias [Title/Abstract] OR Paraphrenias, Involutional [Title/Abstract] OR Psychosis, Involutional [Title/Abstract] OR Involutional Psychoses [Title/Abstract] OR Involutional Psychosis [Title/Abstract] OR Psychoses, Involutional [Title/Abstract] OR Depression, Involutional [Title/Abstract] OR Involutional Depression [Title/Abstract] OR Melancholia, Involutional [Title/Abstract] OR Involutional Melancholia [Title/Abstract]
#3	#1 OR #2
#4	Medicine, Chinese Traditional [MeSH]
#5	Traditional Chinese Medicine [Title/Abstract] OR Traditional Medicine, Chinese [Title/Abstract] OR Zhong Yi Xue [Title/Abstract] OR Chinese Traditional Medicine [Title/Abstract] OR Chinese Medicine, Traditional [Title/Abstract]
#6	#4 OR #5
#7	randomized controlled trial [Publication Type]
#8	randomized [Title/Abstract] OR randomly [Title/Abstract] OR random allocation [Title/Abstract]
#9	#7 OR #8
#10	#3 AND #6 AND #9

### 2.2 Selection criteria

#### 2.2.1 Inclusion criteria

##### 2.2.1.1 Study design

The study included in the NMA is randomized controlled trials (RCTs) for adult MDD.

##### 2.2.1.2 Participants

Participants are adults (older than 18 years old, regardless of gender and race)with a diagnosis of MDD according to The Diagnostic and Statistical Manual of Mental Disorders (DSM-V), International Classification of Diseases (ICD-10) and Chinese Classification of Mental Disorders (CCMD-3).

##### 2.2.1.3 Intervention and comparator

The control group receives western medicine treatment alone. The experimental group uses western medicine at the same time as traditional Chinese medicine (TCM). Note that the western medicine used in the experimental group should be the same as the control group, and there is no limit on the dose and treatment time of TCM.

##### 2.2.1.4 Outcomes

Hamilton Depression Scale (HAMD) score, Symptom Self-Assessment List (SCL-20) score, Beck Depression Inventory (BDI) score are used to evaluate the therapeutic effect. The treatment discontinuation rate for any reason will used to assess acceptability.

#### 2.2.2 Exclusion criteria

We will exclude the following literature: participants with bipolar disorder, psychotic depression, or treatment-resistant depression; the treatment measures in the literature involve other treatments, such as psychological intervention, acupuncture and moxibustion, acupoint sticking, etc., which may affect the interpretation of causal relationship; literature with incomplete data; duplicate published literature.

### 2.3 Data extraction

Two reviewers will extract data from the eligible research back-to-back and conduct cross-checking. In case of disagreement, another reviewer will assist in the discussion of judgment.

#### 2.3.1 Literature sources

The title of the study, name of the first author, contact information, publication year, country of publication, journal.

#### 2.3.2 Methods

Research design type, random sequence generation method, the implementation of allocation concealed, the choice of blind methods, the situation of follow-up and loss of follow-up, selective reporting.

#### 2.3.3 Participants

The total number of participants, age, sex, country, setting, diagnostic criteria, course of the disease, complications.

#### 2.3.4 Interventions

The total number of intervention and comparison groups, including the number of participants, lost follow-up and analysts, the intervention measures and specific methods of intervention and comparison groups, the route of administration, administration time, dosage and course of treatment.

#### 2.3.5 Outcomes

Primary outcome indicators: Effectiveness and acceptability. The efficacy is assessed using the Hamilton Depression Scale (HAMD), and the HAMD score reduction rate ≥50% compared with the baseline is defined as effective. Acceptability is measured by the incidence of adverse reactions.

Secondary outcome indicators: Hamilton Depression Scale (HAMD) score, Symptom Self-Assessment List (SCL-20) score, Beck Depression Inventory (BDI) score, Adverse Reaction Scale (TESS) score.

Record the outcome index at 8 weeks of treatment. If there is no information at 8 weeks of treatment, the outcome index closest to 8 weeks will be recorded; if the time interval from 8 weeks is equal, we will use the results of long-term research.^[16]^

### 2.4 Quality assessment

Two independent analysis for the risk of bias will be performed in strict accordance with the Cochrane Collaboration's risk of bias tool (Cochrane ROB) by 2 reviewers.^[24]^ Disagreement will be discussed and resolved by another reviewer. The assessment content mainly includes the following 6 aspects: selection bias, performance bias, detection bias, attrition bias, reporting bias, and other potential bias. For each index, “low bias risk”, “uncertainty of bias risk” and “high bias risk” are used to judge.

### 2.5 Data analysis

#### 2.5.1 Network meta-analyses

We will select the random effect model. Markov Chain Monte Carlo (MCMC) algorithm will be used to conduct an NMA under the Bayesian framework. With the help of Winbugs 1.4.3 software, through Gibbs sampling, 3 Markov chains are generated, and the number of iterations permitted is 50,000 times (the first 20,000 times are used for the annealing algorithm, the last 30,000 times for sampling). Use STATA15.0 software to perform and present graphical analysis. Dichotomous outcomes use odds ratios (ORs) as the effect indicator, and continuous outcomes use standardised mean differences (SMD) as the effect indicator. Each effect size is given its point estimate and corresponding 95% confidence interval (CI). Calculate surface under the cumulative ranking area (SUCRA) and rank each treatment strategies according to the calculated values. A larger area under the curve indicates the better efficacy of a treatment strategy.

#### 2.5.2 Assessment of heterogeneity, transitivity, consistency

Qualitative heterogeneity will be assessed by Cochrane Q test (χ^2^), and the significance level is set to 0.1. In addition, the *I*-squared (I^2^) test will be used for quantitative statistics of heterogeneity, and the cut-off value is set to 50%.

Describe the distribution of clinical and methodological variables by constructing box diagrams to evaluate transitivity.^[16]^

The node-splitting approach will be used to assess the consistency between direct and indirect evidence, and the significance level is set to 0.05.

#### 2.5.3 Subgroup analysis and sensitivity analysis

The 2 methods are mainly used to perform the robustness test. We will use baseline depression severity, dose, dosage form, study duration, funding source, country for subgroup analysis. For sensitivity analysis, we will use an exclusion method to assess whether the heterogeneity changes with the deletion of a particular study. The excluded studies include the following types: studies with low methodologic quality, small sample size, only published data and unacceptable dosing dose.

#### 2.5.4 Assessment of publication bias

If there is enough research, we will assess the potential publication bias by constructing comparison-adjusted funnel plot and observing its symmetry.

#### 2.5.5 Assessment of evidence quality

Grading of Recommendations Assessment, Development and Evaluation (GRADE) will be used to assess the quality of the evidence.^[25]^ The GRADE offers 5 respects for the assessment: study limitations, inconsistency, imprecision, indirectness, and publication bias. The quality of evidence will be divided into 4 grades: very low, low, moderate and high.

## 3 Discussion

As a treatment strategy with a history of more than 2000 years, TCM provides valuable clinical experience based on long medical practice, which can offer powerful leads for disease treatment.^[26]^ In addition to the traditional decoction, with the strict supervision of the National Drug Regulatory Authority and scientific preparation process, the Chinese medicine decoction pieces have also been made into ointments, pills, granules, capsules and other TCM dosage forms, expanding the scope of application of TCM. The study hopes to summarize the available evidence of TCM for MDD through a comprehensive and systematic literature search. Under the Bayesian framework, objective evaluation is made on the efficacy and acceptability of TCM in the treatment of MDD, and the SUCRA is used to understand the ranking of various TCM strategies more intuitively. Considering that the quality of the analysis may be limited by the quality of the original research data, we will conduct bias risk assessment through Cochrane ROB, credibility assessment through GRADE and consider the potential sources of heterogeneity. At the same time, multi-centre, large-sample, high-quality RCTs are still needed for further analysis in order to help clinicians and medical policymakers make better choices in the treatment of MDD and improve clinical decision-making.

## Author contributions

**Conceptualization:** Yuze Shao, Zhonglin Wang.

**Data curation:** Yuze Shao, Xiaowen Yu.

**Methodology:** Yuze Shao, Zhenyuan Jiang, Zhonglin Wang.

**Project administration:** Yuze Shao, Zhonglin Wang.

**Search strategy:** Yuze Shao, Kai Wang, Wenrong An.

**Statistical analysis:** Yuze Shao, Kai Wang, Yue Han.

**Software:** Yuze Shao, Kai Wang, Xiaowen Yu.

**Writing – original draft:** Yuze Shao, Kai Wang.

**Writing – review & editing:** Yuze Shao, Zhonglin Wang.

## References

[1] Madeira C Vargas-Lopes C Brandão CO Elevated glutamate and glutamine levels in the cerebrospinal fluid of patients with probable alzheimer's disease and depression. Front Psychiatry 2018;9:561. 30459657 10.3389/fpsyt.2018.00561 PMC6232456

[2] Zhou J Zhang C Wu X Identification of genes and pathways related to atherosclerosis comorbidity and depressive behavior via RNA-seq and bioinformation analysis in ApoE (-/-) mice. Ann Translat Med 2019;7:733. 10.21037/atm.2019.11.118 PMC6990041 32042749

[3] Malhi GS Mann JJ Depression. Lancet (London, England) 2018;392:2299–312. 10.1016/S0140-6736(18)31948-2 30396512

[4] Sramek JJ Murphy MF Cutler NR Sex differences in the psychopharmacological treatment of depression. Dialogues Clin Neurosci 2016;18:447–57. 28179816 10.31887/DCNS.2016.18.4/ncutler PMC5286730

[5] Flint J Kendler KS The genetics of major depression. Neuron 2014;81:484–503. 24507187 10.1016/j.neuron.2014.01.027 PMC3919201

[6] Ménard C Pfau ML Hodes GE Immune and neuroendocrine mechanisms of stress vulnerability and resilience. Neuropsychopharmacology 2017;42:62–80. 27291462 10.1038/npp.2016.90 PMC5143517

[7] Dantzer R Neuroimmune interactions: from the brain to the immune system and vice versa. Physiol Rev 2018;98:477–504. 29351513 10.1152/physrev.00039.2016 PMC5866360

[8] Slyepchenko A Maes M Jacka FN Gut microbiota, bacterial translocation, and interactions with diet: pathophysiological links between major depressive disorder and non-communicable medical comorbidities. Psychotherapy Psychosomatics 2017;86:31–46. 27884012 10.1159/000448957

[9] Gassen NC Hartmann J Zschocke J Association of FKBP51 with priming of autophagy pathways and mediation of antidepressant treatment response: evidence in cells, mice, and humans. PLoS Med 2014;11:e1001755. 25386878 10.1371/journal.pmed.1001755 PMC4227651

[10] Kupfer DJ Frank E Phillips ML Major depressive disorder: new clinical, neurobiological, and treatment perspectives. Lancet (London, England) 2012;379:1045–55. 10.1016/S0140-6736(11)60602-8 PMC3397431 22189047

[11] Global Burden of Disease Study 2013 Collaborators. Global, regional, and national incidence, prevalence, and years lived with disability for 301 acute and chronic diseases and injuries in 188 countries, 1990–2013: a systematic analysis for the Global Burden of Disease Study 2013. Lancet (London, England) 2015;386:743–800. 10.1016/S0140-6736(15)60692-4 PMC4561509 26063472

[12] Chisholm D Sweeny K Sheehan P Scaling-up treatment of depression and anxiety: a global return on investment analysis. Lancet Psychiatry 2016;3:415–24. 27083119 10.1016/S2215-0366(16)30024-4

[13] Teply RM Packard KA White ND Treatment of depression in patients with concomitant cardiac disease. Prog Cardiovasc Dis 2016;58:514–28. 26562328 10.1016/j.pcad.2015.11.003

[14] Pineda EA Hensler JG Sankar R Interleukin-1β causes fluoxetine resistance in an animal model of epilepsy-associated depression. Neurotherapeutics 2012;9:477–85. 22427156 10.1007/s13311-012-0110-4 PMC3337012

[15] Roose SP Treatment of depression in patients with heart disease. Biological Psychiatry 2003;54:262–8. 12893102 10.1016/s0006-3223(03)00320-2

[16] Cipriani A Furukawa TA Salanti G Comparative efficacy and acceptability of 21 antidepressant drugs for the acute treatment of adults with major depressive disorder: a systematic review and network meta-analysis. Lancet (London, England) 2018;391:1357–66. 10.1016/S0140-6736(17)32802-7 PMC5889788 29477251

[17] Mutz J Vipulananthan V Carter B Comparative efficacy and acceptability of non-surgical brain stimulation for the acute treatment of major depressive episodes in adults: systematic review and network meta-analysis. BMJ (Clinical research ed) 2019;364:l1079. 10.1136/bmj.l1079 PMC6435996 30917990

[18] Miller KJ Gonçalves-Bradley DC Areerob P Comparative effectiveness of three exercise types to treat clinical depression in older adults: a systematic review and network meta-analysis of randomised controlled trials. Ageing Res Rev 2020;58:100999. 31837462 10.1016/j.arr.2019.100999

[19] Zhou XD Shi DD Zhang ZJ Antidepressant and anxiolytic effects of the proprietary Chinese medicine Shexiang Baoxin pill in mice with chronic unpredictable mild stress. J Food Drug Anal 2019;27:221–30. 30648575 10.1016/j.jfda.2018.08.001 PMC9298624

[20] Zhu HZ Liang YD Ma QY Xiaoyaosan improves depressive-like behavior in rats with chronic immobilization stress through modulation of the gut microbiota. Biomed Pharmacother 2019;112:108621. 30798141 10.1016/j.biopha.2019.108621

[21] Liu X Zheng X Du G Brain metabonomics study of the antidepressant-like effect of Xiaoyaosan on the CUMS-depression rats by (1)H NMR analysis. J Ethnopharmacol 2019;235:141–54. 30708033 10.1016/j.jep.2019.01.018

[22] Yan L Hu Q Mak MS A Chinese herbal decoction, reformulated from Kai-Xin-San, relieves the depression-like symptoms in stressed rats and induces neurogenesis in cultured neurons. Sci Rep 2016;6:30014. 27444820 10.1038/srep30014 PMC4957105

[23] Shamseer L Moher D Clarke M Preferred reporting items for systematic review and meta-analysis protocols (PRISMA-P) 2015: elaboration and explanation. BMJ (Clinical Research ed) 2016;354:i4086. 10.1136/bmj.i4086 27444514

[24] Higgins JPT Cochrane Handbook for Systematic Reviews of Interventions Version 5.0.1. Naunyn-Schmiedebergs Archiv für experimentelle Pathologie und Pharmakologie, 2008, 5:S38.

[25] Guyatt G Oxman AD Akl EA GRADE guidelines: 1. Introduction-GRADE evidence profiles and summary of findings tables. J Clin Epidemiol 2011;64:383–94. 21195583 10.1016/j.jclinepi.2010.04.026

[26] Liu T Ding Y Wen A Traditional Chinese medicine for ischaemic stroke. Lancet Neurol 2018;17:745. 10.1016/S1474-4422(18)30290-4 30129474

